# Carvacrol Loaded Solid Lipid Nanoparticles of Propylene Glycol Monopalmitate and Glyceryl Monostearate: Preparation, Characterization, and Synergistic Antimicrobial Activity

**DOI:** 10.3390/nano9081162

**Published:** 2019-08-14

**Authors:** Junbo He, Shuangshuang Huang, Xiaotao Sun, Lijuan Han, Chao Chang, Weinong Zhang, Qixin Zhong

**Affiliations:** 1 Key Laboratory for Deep Processing of Major Grain and Oil, Ministry of Education, College of Food Science & Engineering, Wuhan Polytechnic University, Wuhan 430023, China; 2 Hubei Key Laboratory for Processing and Transformation of Agricultural Products, Wuhan Polytechnic University, Wuhan 430023, China; 3 Beijing Key Lab of Flavor Chemistry, Beijing Technology and Business University, Beijing 100048, China; 4 Department of Food Science, The University of Tennessee, Knoxville, TN 37996, USA

**Keywords:** solid lipid nanoparticles, carvacrol, propylene glycol monopalmitate, synergistic antimicrobial activity

## Abstract

To develop solid lipid nanoparticles (SLNs) with stable lipid matrix structures for the delivery of bioactive compounds, a new class of SLNs was studied using propylene glycol monopalmitate (PGMP) and glyceryl monostearate (GMS) mixtures and carvacrol as a model lipophilic antimicrobial. Stable SLNs were fabricated at PGMP:GMS mass ratios of 2:1 and 1:1, and the carvacrol loading was up to 30% of lipids with >98% encapsulation efficiency and absence of visual instability. Fluorescence spectra and release profiles indicated the carvacrol was successfully encapsulated and homogeneously distributed within the SLNs. SLNs fabricated with equal masses of PGMP and GMS had better stability of carvacrol during storage and higher sphericity than those with a ratio of 2:1 and were much more effective than free carvacrol against *Escherichia coli* O157:H7 and *Staphylococcus aureus*. These findings demonstrated the potential applications of the studied SLNs in delivering lipophilic bioactive compounds in food and other products.

## 1. Introduction

Solid lipid nanoparticles (SLNs) are a class of colloidal particles composed of solid lipids at the application temperature and have become a group of fascinating delivery vehicles in food, cosmetic, and medical sciences owing to the good biocompatibility [1,2,3]. Encapsulation of bioactive compounds, particularly poorly water-soluble or unstable ones, in SLNs can improve water-solubility of these compounds, as well as stability against oxidative, UV, and thermal stresses [4]. However, low loading capacity and polymorphic transformation during storage are two major drawbacks of SLNs greatly limiting their application [5]. Especially, the polymorphism transformation of lipid matrixes in SLNs results from rearrangement of crystalline lattices to form thermodynamically more stable polymorph, e.g., from α- to β-form crystals, and the resultant increase in lipid molecular ordering causes the expulsion of initially incorporated bioactive compounds [6,7]. Maintaining the crystalline structure or slowing the polymorphic transition is needed to overcome the challenges of SLNs as delivery systems. We recently reported that the SLNs prepared with a diacylglycerol, 1-laurin-3-palmitin, had a higher loaded capacity and better stability than those of glyceryl monostearate (GMS) and glyceryl tripalmitate due to the stable, mostly β-form, crystalline polymorph of 1-laurin-3-palmitin [8]. Therefore, identifying solid food lipids forming stable crystalline structures is a new direction to study SLNs for food application.

Propylene glycol monoesters (PGMEs) are widely used as food emulsifiers for cake mixes, sponge cakes, whipped toppings, and bread [9]. Furthermore, the purified PGMEs have specific crystalline properties with the ability to stabilize the meta-stable α-form crystals of distilled GMS even for a year, and this kinetic stability meets the shelf-life of many aerated products [9,10,11]. The kinetically stable α-form crystals formed of the mixture of PGME and GMS, also referred to as “conjoined crystals”, provide a rational lipid matrix to fabricate SLNs. However, there is currently no study on SLNs prepared with PGME and GMS mixtures.

Antimicrobials are one group of bioactive compounds that can utilize the characteristics of SLNs as delivery systems, such as the release kinetics controllable by lipid matrix structures. Carvacrol (5-isopropyl-2-methylphenol) is a major phenolic constituent of essential oils derived from thyme, marjoram, and oregano [12]. Carvacrol possesses broad biological activities, such as antioxidant [13,14], antimicrobial [14,15,16], and antitumor activities [17]. As generally recognized as safe (GRAS) food additive, carvacrol has been widely used as a flavoring or antimicrobial agent in the food industry. However, the utilization of carvacrol faces some challenges including its poor-water solubility, strong odor, and instability when exposed to air, light, or heat. Encapsulation of carvacrol to increase the water solubility or stability has been reported for systems such as chitosan-based nanoparticles [18], β-cyclodextrin inclusion complexes [19], nanoemulsions [20], and zein nanoparticles [21]. However, SLNs are rarely studied to encapsulate carvacrol to utilize advantages of SLNs as delivery systems.

The first objective of the present study was to investigate the mixture of propylene glycol monopalmitate (PGMP) and GMS as novel lipid matrixes for fabricating of SLNs and the properties of encapsulating carvacrol. The second objective was to study the antimicrobial activity of carvacrol-loaded SLNs (Car-SLNs). The Car-SLNs were characterized for dimension using dynamic light scattering (DLS), zeta-potential, fluorescence spectra, release profile, and morphology using transmission electron microscopy (TEM). The antimicrobial activities of Car-SLNs were evaluated against model Gram-negative bacterium *Escherichia coli* O157:H7 and Gram-positive bacterium *Staphylococcus aureus*.

## 2. Materials and Methods

### 2.1. Materials

1,2-Propylene glycol (>99% pure), palmitoyl chloride (>97% pure), carvacrol (99% pure), and Tween 80 (T80) were purchased from Aladdin Bio-Chem Technology Co., Ltd. (Shanghai, China). GMS (analytic reagent) was purchased form Shanpu Chemical Co., Ltd. (Shanghai, China). The bacterial strains of *E. coli* O157:H7 was obtained from the Culture Collection of the College of Food Science and Engineering at Wuhan Polytechnic University and *S. aureus* (CCTCC AB 91093) was purchased from China Center for Type Culture Collection (Wuhan, China). Lysogeny broth (LB) was prepared by dissolving 25 g of powder, purchased from Qingdao Hope Bio-Technology Co., Ltd. (Qingdao, China), in 1000 mL deionized water and autoclaving at 121 °C for 15 min. Double distilled water (ddH_2_O) was used in the preparation of all samples. All other chemicals and solvents were of analytical grade and were used as received.

### 2.2. Synthesis of Propylene Glycol Monopalmitate

PGMP was prepared by the reaction of propylene glycol and palmitoyl chloride in the presence of triethylamine as the base, which is different from the report by the direct esterification method [22]. Briefly, a mixture of 1,2-propylene glycol (10 mmol) and triethylamine (14 mmol) in dichloromethane (30 mL) was cooled to 0 °C in ice-water, and palmitoyl chloride (12 mmol) dissolved in dichloromethane (5 mL) was added dropwise. After stirring at room temperature for 3 h, the mixture was washed with water (30 mL) and then 0.1 M NaOH solution (30 mL). The resulting organic phase was dried by MgSO_4_ overnight, concentrated under reduced pressure to harvest the PGMP as white solids that had a melting point of 42–44 °C and a yield of 88%. Spectroscopic properties of the obtained PGMP powder are studied in detail in the Supporting Information, however, these data were not reported in the published work [22].

### 2.3. Preparation of SLN Dispersions

**Scheme 1:**
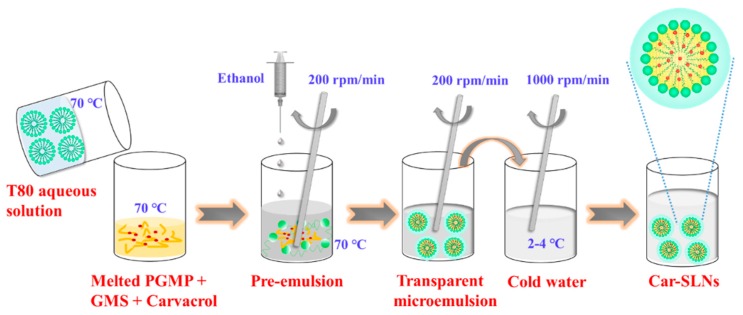
The microemulsion method for the preparation of carvacrol-loaded solid lipid nanoparticles (Car-SLNs).

The SLN dispersions were prepared by a microemulsion template method (Scheme 1) [8]. Briefly, 90 mg of lipids containing the mixture of PGMP and GMS at a mass ratio from 1:0, 2:1, 1:1, 1:2, to 0:1 was melted at 70 °C under continuous stirring. The aqueous phase was prepared by adding T80 (Tween 80, 270 mg) to ddH_2_O (2 g) at 70 °C. The mass ratio of lipid to T80 was fixed at 1:3. Then, the aqueous phase was added rapidly to the lipid phase at 70 °C, after which, ethanol (0.5 mL) was added dropwise to the pre-emulsion and stirred for 5 min to form a transparent microemulsion. The hot microemulsion was subsequently dispersed in 5 parts (*v*/*v*) of cold water (2–4 °C) to solidify lipids. All samples were stored at 25 °C for further analysis.

### 2.4. Determination of Z-Average Mean Diameter, Polydispersity Index (PDI), and Zeta-Potential

The Z-average mean diameter and PDI of the prepared SLN dispersions were determined using DLS (model Zetasizer Nano 90ZS, Malvern Instruments Ltd., Worcestershire, UK). The laser wavelength was set at 633 nm, and the material/dispersant reflective index was 1.590/1.330. Samples were prepared by diluting the SLN dispersions 30-fold using ddH_2_O. The zeta-potential was determined by microelectrophoresis on the same instrument.

### 2.5. Preparation and Characterization of Carvacrol-Loaded SLNs

To prepare Car-SLNs, carvacrol was dissolved at 20%, 30%, and 40% mass of PGMP and GMS mixtures melted at 70 °C, followed by preparing SLNs as above. Loading capacity of carvacrol was evaluated by adding different amounts of carvacrol into the lipids and preparing the SLNs dispersions based on the optimized formula. The Z-average mean diameter, PDI, and zeta-potential of prepared Car-SLNs after storage at 25 °C for 0 and 30 days were determined as in the previous section.

### 2.6. Determination of Entrapment Efficiency of Car-SLNs

Entrapment efficiency (EE) was determined using an ultrafiltration method with some modification [23]. The Car-SLN dispersions were passed through a flat polyethersulfone (PES) ultrafiltration membrane (SEPRO Membranes, Inc., USA) with a molecular weight cur-off (MWCO) of 10 kDa using an ultrafiltration cup (UHP-25K, Advantech Toyo, Tokyo, Japan) under constant nitrogen passage of 0.45 MPa. The free carvacrol in filtrate was analyzed with a HPLC method presented below. The EE was calculated using the following Equation (1):

EE (%) = [(W_t_ − W_f_)/W_t_] × 100%, (1)

where W_t_ and W_f_ are the total mass of carvacrol added and the mass of free carvacrol, respectively.

### 2.7. Chemical Stability of Carvacrol Loaded in SLNs

The chemical stability of carvacrol after storage at 25 °C for seven and 30 days was evaluated to investigate the protection effect of SLNs. To quantify residual carvacrol, 200 µL of a Car-SLN dispersion was dissolved in dry methanol (2 mL) and sonicated for 30 min, and then 200 µL of the mixture was diluted by 10 times using methanol and used for HPLC analysis. The Waters 1525 HPLC system (Waters Corporation, Milford, MA, USA) was equipped with a UV-vis detector at 274 nm and an Agilent C18 column (250 mm × 4.5 mm, 5 µm) kept at 30 °C. The mobile phase was composed of acetonitrile and deionized water (60:40, *v*/*v*) and run at a flow rate of 1 mL/min during isocratic separation. The carvacrol content was determined by referring to a calibration curve established with standard solutions of carvacrol dissolved in methanol (*R*^2^ = 0.998). The chemical stability was determined by calculating the percentage of residual carvacrol with reference to that on day 0.

### 2.8. Fluorescence Study of Car-SLN Dispersions

The interaction between carvacrol and SLNs was studied with fluorescence spectrophotometry (model F-4600, Hitachi, Ltd., Tokyo, Japan). Car-SLN dispersions were diluted by nine times with ddH_2_O to fit the instrument sensitivity and the fluorescence spectra were obtained at an excitation wavelength of 227 nm and an emission wavelength range from 200 to 500 nm. The slit width was 5 nm for both excitation and emission. Carvacrol pre-dissolved in ethanol and ddH_2_O (0.3 mg/mL), and SLN dispersions without carvacrol were studied as controls.

### 2.9. Release Kinetics of Carvacrol from the SLNs

The release kinetics of carvacrol in SLNs was evaluated using a dialysis membrane method [24]. Briefly, the freshly prepared Car-SLN dispersion was diluted with release medium (water/ethanol, 9:1 *v*/*v*) to a carvacrol amount of 2.0 mg. The membrane bag (molecular weight cut-off of 3500 Da) filled with 10 mL of the diluted Car-SLN dispersion was immersed in 400 mL of the release medium that was stirred at 100 rpm and 25 °C. Two milliliter aliquots of the release medium were withdrawn at predetermined time intervals and the same amount of the release medium was replenished. The carvacrol in the collected samples was determined using the above HPLC protocol. The release kinetics of free carvacrol was performed in the same manner for 10 mL of 2.0 mg carvacrol dissolved in ethanol. All measurements were performed in three replicates.

### 2.10. Transmission Electron Microscopy

The morphology of SLNs with the carvacrol loading of 30% in the lipid phase after 30 days storage at 25 °C was evaluated using a Hitachi HT7700 transmission electron microscope (Hitachi High-Technologies, Pleasanton, CA, USA). The Car-SLN dispersion was initially deposited onto a 200-mesh copper grid and allowed to dry for 15 min. After drying, the sample negatively stained with 2% phosphotungstic acid was analyzed at a voltage of 80 kV.

### 2.11. Antimicrobial Activity of Car-SLNs

The minimum inhibitory concentration (MIC) and minimum bactericidal concentration (MBC) of free carvacrol, bare SLNs, and Car-SLNs with 30% loading against both Gram-negative *E. coli* O157:H7 and Gram-positive *S. aureus* were determined by the microbroth dilution method according to a previous study with slight modification [25]. Free carvacrol stock solution was prepared by diluting the carvacrol solution (2 mg/mL in ethanol) to 1 mg/mL with LB. From the stock solution, the working solutions were prepared by further diluting the stock solution using LB to 0.1, 0.2, 0.25, 0.3, 0.4, 0.5, 0.6, 0.7, 0.8, and 1 mg/mL of carvacrol. Car-SLNs dispersions were directly diluted to the same concentrations of free carvacrol with LB. To wells of sterile 96-well microtiter plates were first added 120 µL of bacterial culture (ca. 10^6^ CFU/mL bacteria) followed by 120 µL of the antimicrobial sample. Absorbance of wells at 630 nm was measured using an automated microplate reader (PerkinElmer EnSpire, Waltham, MA, USA) before and after 24 h incubation at 37 °C. The MIC was defined as the carvacrol concentration that allowed less than or equal to a 0.05 increase in absorbance after 24 h incubation. To determine MBC, 20 µL of the mixture from wells showing no growth was spread on LB plates. Following incubation of LB plates at 37 °C for 24 h, the lowest carvacrol concentration corresponding to no bacterial growth was defined as the MBC. The bare SLN dispersions were used as a control and diluted as the Car-SLNs in the assay.

### 2.12. Statistical Analysis

All experiments were performed in triplicate. The statistical analyses were conducted using SPSS software (IBM SPSS statistic 19) with one-way ANOVA and Tukey’s multiple comparison test to determine the difference among samples. The significant level (*p*) was set as 0.05.

## 3. Results and Discussion

### 3.1. Synthesis of PGMP

To form kinetically stable α-form crystals, the purity of PGMEs needs to be 90% or higher [10,11]. The purity of PGMP synthesized in the present study was evaluated using ^1^H NMR, and no propylene glycol dipalmitate was detected (Supplementary Figure S1). The high purity of PGMP can be attributed to the steric effect that the formation of PGMP hinders the further esterification with another palmitoyl chloride molecule. The FT-IR spectra of PGMP (Supplementary Figure S2) presented in the supporting information revealed typical characteristic peaks, such as the broad peak at 3404 cm^−1^ (stretching vibration of OH), sharp peak at 1743 cm^−1^ (stretching vibration of esterified carbonyl group), sharp peak at 1460 cm^−1^ (bending vibration of methylene group), and sharp peak at 725 cm^−1^ (rocking vibration of long methylene chain). The mass spectrum of PGMP (Supplementary Figure S3) was observed at the *m*/*z* of 337.37 ([M + Na]^+^). Therefore, the synthesized PGMP had a sufficiently high purity to prepare SLNs.

### 3.2. Effect of the Mass Ratio of PGMP and GMS on SLN Formation

To construct stable SLNs dispersions, the mass ratio of PGMP and GMS was optimized firstly using T80 as a surfactant at the one-third mass of solid lipids (PGMP + GMS). The Z-average mean diameter, PDI, zeta-potential, and visual stability of SLN dispersions prepared by using different PGMP:GMS mass ratios are summarized in Table 1. With the increase of the PGMP:GMS mass ratio from 1:0 to 1:1, the Z-average mean diameter, PDI, and zeta-potential magnitude of these SLNs dispersions all consistently increased. The PDI was all smaller than 0.3, indicating relatively monodispersed SLNs. The impact of lipid composition on Z-average mean diameter can be attributed to the polarity and melting point/viscosity of lipids during microemulsion formation. PGMP has a hydrophile–lipophile balance (HLB) value (4.6) higher than that of GMS (3.6) calculated according to Equation (2). An HLB value of the lipid phase closer to that of surfactant (15.0 for T80) lowers the interfacial tension and facilitates formation of smaller lipid droplets in microemulsions [26]. An increase in GMS content lowers the HLB of lipid mixture to be further away from that of T80 and therefore is expected to increase the dimension of SLNs, which agrees with the data in Table 1. A higher melting point/viscosity of GMS (58–60 °C) than PGMP (42–44 °C) [27] increases the interfacial tension and contributes to the increase in droplet size of microemulsions.

HLB = 20 × [M_Hydrophilic_/(M_Hydrophilic_ + M_Lipophilic_)], (2)

where M_Hydrophilic_ and M_Lipophilic_ are the molecular mass of hydrophilic and lipophilic (palmitate or stearate) portions of a surfactant [28].

Visual stability of SLNs is also summarized in Table 1. SLNs prepared with PGMP:GMS mass ratios of 2:1 and 1:1 were still stable after one month, whereas those with PGMP only precipitated after three days and those with PGMP:GMS mass ratios of 1:2 and 0:1 showed precipitation after one day. PGMP:GMS mass ratios of 2:1 and 1:1 were chosen to prepare Car-SLNs.

### 3.3. Properties of SLNs Encapsulating Carvacrol

**Figure 1:**
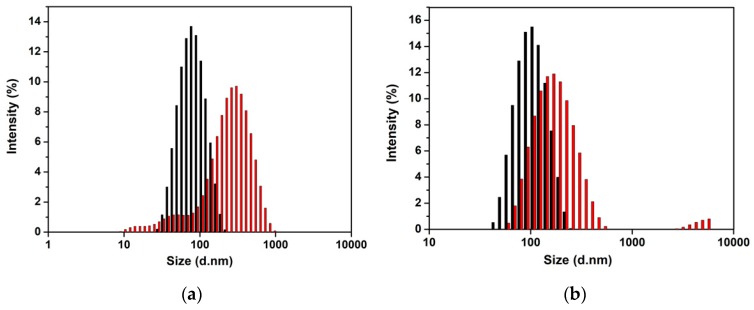
Intensity size distributions of SLNs prepared with a PGMP:GMS mass ratio of 2:1 (**a**) or 1:1 (**b**) and a carvacrol loading at 30% mass of lipids, before (black column) and after (red column) 30-day storage at 25 °C.

On the basis of the optimized formulations for bare SLNs dispersions, carvacrol was encapsulated in the SLNs with PGMP:GMS mass ratios of 2:1 and 1:1. Effects of loading capacities of carvacrol on the Z-average mean diameter, PDI, zeta-potential, and EE were investigated and the results are shown in Table 2. The EEs were all higher than 98%. The Z-average mean diameters of freshly prepared Car-SNLs using the PGMP:GMS mass ratio of 2:1 were smaller than those with the PGMP:GMS mass ratio of 1:1, showing the same trend as the bare SLNs (Table 1). The significant growth of the nanoparticle dimension was observed after 30-day storage, more significant at a PGMP:GMS mass ratio of 2:1 than at 1:1, suggesting particle aggregation. After storage for 30 days, SLNs with a PGMP:GMS mass ratio of 2:1 showed the appearance of smaller particles, in addition to shifting to bigger particles (Figure 1a), which is the characteristics of Ostwald ripening [29]. Physically, as carvacrol has a solubility of 0.45 mg/mL in water and 1.63 mg/mL in 5% aqueous ethanol and carvacrol in smaller particles has a higher solubility [29,30], carvacrol in smaller particles will dissolve into the continuous phase, and the dissolved carvacrol will join in bigger droplets to lower system free energy. As a result, smaller particles become smaller, while bigger particles become bigger after storage. In contract, SLNs prepared with a PGMP:GMS mass ratio of 1:1 showed the shifting to bigger particles and an appearance of big particles of one order of magnitude higher (Figure 1b), which may indicate particle aggregation. As the zeta-potential magnitude of SLNs is smaller than 20 (Table 2), the electrostatic repulsion may not be strong enough to prevent particle aggregation [31]. The absence of Ostwald ripening for SLNs at a PGMP:GMS ratio of 1:1 may result from the higher melting point (mass transfer resistance for carvacrol) of the lipid matrix than SLNs at a PGMP:GMS ratio of 2:1, as discussed previously. The aggregation and/or Ostwald ripening resulted in the widened particle size distribution (Figure 1) and increased PDI (Table 2) after storage. Nevertheless, the z-average mean diameter of all treatment was smaller than 200 nm after storage and all SLNs remained visually stable, except for minor precipitation observed at the highest carvacrol level treatments.

### 3.4. Chemical Stability of Carvacrol in SLNs

Carvacrol as a natural bioactive compound is chemically unstable since it is sensitive to light and oxygen [32]. Therefore, the chemical stability of carvacrol was evaluated over 30 days. The results are shown in Table 3. Free carvacrol dissolved in ethanol to a concentration equivalent to the Car-SLNs with a carvacrol loading at 30% mass of lipids showed the reduction to 84.1% and 80.0% after seven and 30 days of storage, respectively. Nanoencapsulation of carvacrol in SLNs improved the chemical stability. Car-SLNs fabricated with a PGMP:GMS mass ratio of 1:1 displayed a better stability (>95% residual carvacrol) than those of a PGMP:GMS mass ratio of 2:1 (~92%) after seven-day storage. The same trend was observed on day 30, and the higher residual carvacrol% in Car-SLNs was observed at a lower carvacrol loading. As minor precipitation was observed for SLNs prepared with a carvacrol loading at 40% mass of lipids, the optimum carvacrol loading capacity at the studied conditions was concluded for 30% of the lipids, which was used in subsequent studies.

The chemical stability of carvacrol in SLNs may be related to the crystal stability. Previous studies indicated that an equal molar ratio of PGME and GMS was the most effective mixture for retaining α-form crystals, while other molar ratios resulted in the polymorphic transformation to the more stable high-melting β-form crystals [10]. In this study, SLNs with equal masses of PGMP (MW = 314 Da) and GMS (MW = 358 Da) had a molar ratio of almost 1:1. Therefore, the SLNs formulated with a PGMP:GMS mass ratio of 1:1 displayed better carvacrol stability during storage, possibly attributed to the less expulsion of carvacrol due to the stable α-form crystals than those with a PGMP:GMS mass ratio of 2:1.

### 3.5. Interactions between Lipids and Carvacrol Studied with Fluorescence Spectroscopy

**Figure 2:**
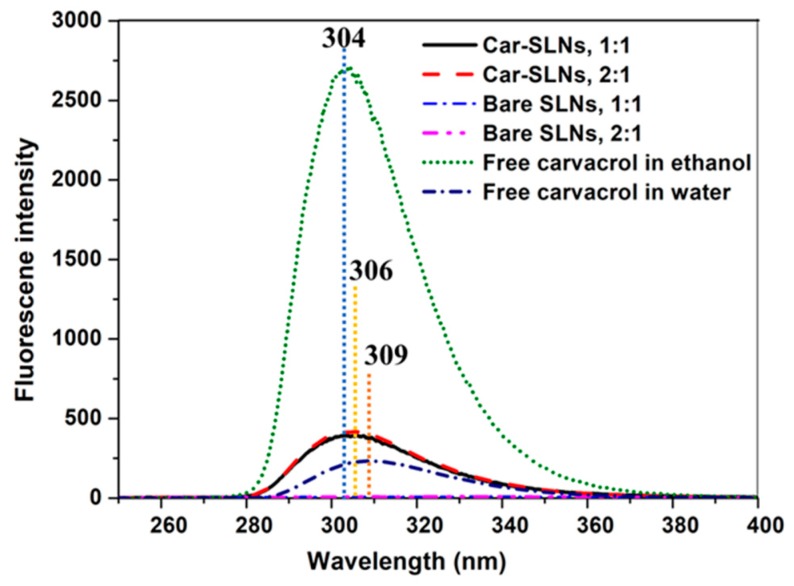
Fluorescence spectra of Car-SLNs with a carvacrol loading at 30% mass of lipids and a PGMP:GMS mass ratio of 2:1 or 1:1 and the corresponding bare SLNs without carvacrol, and carvacrol pre-dissolved in ethanol and water.

Fluorescence spectroscopy was used to investigate the interaction of carvacrol with SLNs, with comparison to the respective bare SLN dispersions and free carvacrol. Figure 2 shows strong fluorescence intensity of carvacrol dissolved in ethanol, with the maximum at a wavelength of 304 nm; whereas carvacrol dispersed in water had much weaker intensity and the wavelength of maximum intensity shifted to 309 nm. These results indicated that the intrinsic fluorescence intensity of carvacrol was affected by its environmental polarity and an increase in polarity of environment lowered the fluorescence intensity and resulted in the red-shift of the maximum intensity wavelength. The observation agreed with the literature, as the reduced solubility in more polar water than in ethanol weakens the exposure of carvacrol for excitation by UV radiation [33].

The fluorescence intensity of Car-SLNs was also much lower than carvacrol dissolved in ethanol but was higher than carvacrol dispersed in water (Figure 2). Dispersion in SLNs with a small dimension (Table 2), i.e., a large surface area, likely increased the exposure than dispersing in water, but not to an extent as carvacrol molecularly dissolved in ethanol [34]. Bare SLNs without carvacrol had insignificant fluorescence, and Car-SLNs with same carvacrol loading but different PGMP:GMS mass ratios thus had similar fluorescence spectra (Figure 2). The fluorescence spectra may also provide some insights about structures of SLNs that have an overall surfactant shell and lipid core structure, and the lipid matrix can have a carvacrol core-lipid shell, lipid core-carvacrol shell, or homogeneous distribution structure [35]. If carvacrol is predominantly present in the outer shell of the lipid matrix, carvacrol will have high possibility to interact with water to cause the fluorescence quenching by water. Likewise, the predominant presence of carvacrol in the lipid matrix core may have the limited excitation by UV. The fluorescence intensity of carvacrol in SLNs being between that dissolved in ethanol and water suggests that carvacrol should be homogeneously dispersed in the SNLs, as proposed in Scheme 1.

### 3.6. Release Profile of Carvacrol in SLNs

**Figure 3:**
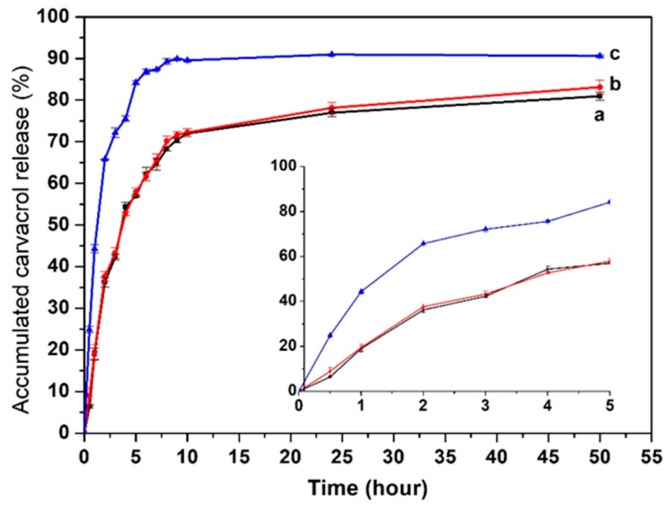
Release profiles of carvacrol from SLNs with a carvacrol loading at 30% mass of lipids and a PGMP:GMS mass ratio of 2:1 (**b**) or 1:1 (**a**), with comparison to free carvacrol dissolved in ethanol (**c**). The inset figure shows release kinetics in the first 5 h. The released carvacrol was detected in the release medium outside the dialysis bag. Error bars are standard derivations (*n* = 3).

Release kinetics was established to obtain further information about the interaction between carvacrol and other SLN components, as well as mechanisms involved in the carvacrol release. Figure 3 shows that encapsulation of carvacrol in SLNs altered the release profile, showing a slower rate initially than that of free carvacrol. For free carvacrol, 50% release was obtained in 1.5 h, and the osmotic equilibrium was achieved after 10 h with 90% release. When carvacrol was encapsulated in SLNs, 50% release of carvacrol was obtained in 6 h, followed by a smaller rate to 80% release after 50 h. The release profile of bioactive compounds in SLNs always followed biphasic pattern, the initial burst release and prolonged release [36,37,38]. The initial burst release results from the diffusion of carvacrol in the particle surface, and the subsequent prolonged release results from the liberation of carvacrol in the lipid matrix [39]. Therefore, the release profile of carvacrol in SLNs further verified the conclusion that carvacrol located both on the surface and core of SLNs, as previously discussed based on fluorescence spectra.

### 3.7. Morphology of Carvacrol-Loaded SLNs

**Figure 4:**
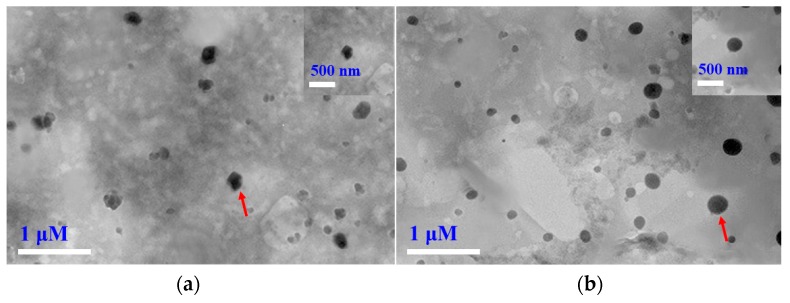
TEM analysis of Car-SLNs prepared with a carvacrol loading at 30% mass of lipids and a PGMP:GMS mass ratio of 2:1 (**a**) or 1:1 (**b**). The particles shown in insert images were pointed by red arrows.

The detailed morphology of Car-SLNs was investigated by TEM (Figure 4). SLNs prepared with PGMP:GMS mass ratios of 2:1 and 1:1 were well dispersed with the size ranging between 100 nm to 200 nm. However, Car-SLNs with a PGMP:GMS mass ratio of 1:1 were more spherical than those with a ratio of 2:1. As previously discussed, the combination of PGME and GMS with an equal molar ratio results in co-crystals with stable polymorph [40], while SLNs with polymorphic transformation from α- to β-form crystals can be evidenced by the formation of irregularly shaped particulates [41].

### 3.8. Antimicrobial Activity

The MIC and MBC of free and nanoencapsulated carvacrol against *E. coli* O157:H7 and *S. aureus* in comparison with the corresponding bare SLNs are summarized in Table 4. The MIC of free carvacrol against *E. coli* O157:H7 and *S. aureus* was 0.250 and 0.125 mg/mL, respectively, which are close to the respective reported range of 0.220–4.880 and 0.171–0.439 mg/mL [42]. The bare SLNs also displayed antimicrobial activities against *E. coli* O157:H7 and *S. aureus* with the MIC values of 0.300 and 0.250 mg/mL at PGMP:GMS mass ratios of 2:1 and 1:1, respectively, and the higher antimicrobial activities of the latter indicated the more significant role of GMS. For the Car-SLNs, a significantly reduced MIC was found both against *E. coli* O157:H7 and *S. aureus* when compared with free carvacrol. In addition, the MBC of Car-SLNs against *E. coli* O157:H7 and *S. aureus* was investigated using sample concentration above MIC values. Car-SLNs showed the MBC values against *E. coli* O157:H7 and *S. aureus* of 0.200 and 0.125 mg/mL at the PGMP:GMS mass ratio of 2:1, and 0.125 and 0.100 mg/mL at the PGMP:GMS mass ratio of 1:1, respectively. These results indicated the synergistic effect of carvacrol and lipid matrix in the antimicrobial activities, with the PGMP:GMS mass ratio of 1:1 possessing more potent synergism. The synergism may be significant to lower the application level of carvacrol to overcome the negative sensory properties of carvacrol used in food preservation [43]. However, sensory studies are to be conducted in the future in specific food matrices.

The interaction of lipophilic carvacrol and phospholipid membrane of microorganisms might cause dramatic changes of the membrane structure [44], which would eventually increase passive permeability of membrane and the measured antimicrobial activity [45,46]. The MIC and MBC values of free carvacrol against *E. coli* O157:H7 were twice of those against *S. aureus* (Table 4). However, after nanoencapsulation of carvacrol in SLNs, the MIC and MBC values against *E. coli* O157:H7 and *S. aureus* became closer (Table 4), indicating that SLNs enhance the permeation of carvacrol in the outer membrane of *E. coli* O157:H7.

## 4. Conclusions

In conclusion, high purity PGMP was synthesized and used as a lipid matrix with GMS to fabricate the Car-SLNs. The 2:1 and 1:1 mass ratio of PGMP:GMS were feasible to prepare stable SLNs at the studied conditions and were capable of loading carvacrol at up to 30% mass of the lipids with an EE of higher than 98%. Although particle growth was observed, Car-SLNs remained smaller than 200 nm and visually stable after 30-day storage. Fluorescence spectra indicated the carvacrol was homogeneously dispersed in the SLNs, which was further supported by the release profile. The high sphericity of Car-SLNs with a PGMP:GMS mass ratio of 1:1 suggested the absence of polymorphic transformation, which is important to SLN physical and chemical stability. The enhanced antimicrobial activities of Car-SLNs against *E. coli* O157:H7 and *S. aureus*, especially those fabricated with equal masses of PGMP and GMS show the potential applications of the studied new class of SLNs for food and cosmetic applications.

## Figures and Tables

**Table 1:** Effects of mass ratio of PGMP and GMS on the Z-average mean diameter (Z-average), polydispersity index (PDI), zeta-potential, and visual stability of solid lipid nanoparticle dispersions.

PGMP:GMS	Z-Average (nm)	PDI	Zeta-Potential (mV)	Visual Stability
1:0	14.9 ± 0.4	0.133 ± 0.017	5.6 ± 0.9	Precipitation after 3 days
2:1	18.6 ± 0.2	0.178 ± 0.008	−11.0 ± 0.1	Stable for 1 month
1:1	25.3 ± 0.7	0.296 ± 0.003	−13.7 ± 0.8	Stable for 1 month
1:2	ND ^1^	ND	ND	Precipitation after 1 day
0:1	ND	ND	ND	Precipitation after 1 day

^1^ ND: Not determined due to precipitation.

**Table 2:** The Z-average mean diameter (Z-average), polydispersity index (PDI), zeta-potential, and encapsulation efficiency (EE%) values for the Car-SLNs dispersions ^1^.

PGMP:GMS	Carvacrol Loading (% Mass of Lipids)	Z-Average (nm)		PDI		Zeta-Potential (mV)		EE (%)
		0 Day	30 Days	0 Day	30 Days	0 Day	30 Days	0 Days
2:1	0	18.6 ± 0.2 ^d^	32.9 ± 0.5 ^d^	0.178 ± 0.008 ^a^	0.292 ± 0.027 ^b^	−11.0 ± 0.1 ^ab^	−14.1 ± 1.6 ^c^	
	20	39.8 ± 0.6 ^c^	149.4 ± 1.8 ^c^	0.184 ± 0.010 ^a^	0.221 ± 0.018 ^c^	−9.73 ± 0.1 ^b^	−18.5 ± 0.7 ^b^	98.0 ± 0.1 ^c^
	30	68.9 ± 0.1 ^b^	159.8 ± 2.5 ^b^	0.182 ± 0.012 ^a^	0.313 ± 0.002 ^a,b^	−12.6 ± 1.7 ^a^	−21.2 ± 1.6 ^a,b^	98.3 ± 0.1 ^a^
	40	95.4 ± 0.7 ^a^	165.8 ± 3.4 ^a^	0.182 ± 0.008 ^a^	0.355 ± 0.051 ^a^	−11. 9± 1.4 ^a,b^	−23.2 ± 2.9 ^a^	98.2 ± 0.2 ^a,b^
1:1	0	25.3 ± 0.7 ^d^	55.1 ± 0.2 ^d^	0.296 ± 0.003 ^a^	0.278 ± 0.003 ^a^	−13.7 ± 0.8 ^b^	−15.3 ± 1.3 ^c^	
	20	56.1 ± 0.3 ^c^	98.7 ± 0.9 ^c^	0.211 ± 0.010 ^c^	0.198 ± 0.002 ^c^	−17.1 ± 0.6 ^a^	−17.4 ± 1.1 ^b^	99.2 ± 0.1 ^b^
	30	89.3 ± 1.2 ^b^	155.2 ± 2.7 ^b^	0.224 ± 0.008 ^c^	0.233 ± 0.004 ^b^	−17.9 ± 1.4 ^a^	−18.6 ± 1.2 ^a,b^	99.4 ± 0.1 ^a^
	40	115.8 ± 0.8 ^a^	193.5 ± 2.5 ^a^	0.239 ± 0.005 ^b^	0.228 ± 0.016 ^b^	−16.6 ± 0.6 ^a^	−19.9 ± 0.5 ^a^	98.1 ± 0.1 ^c^

^1^ Data not sharing the same superscript letter in the same column are significantly different (*p* < 0.05).

**Table 3:** Chemical stability of free carvacrol and carvacrol loaded in SLNs.

PGMP:GMS	Carvacrol Loading (% Mass of Lipids)	Residual Carvacrol (%) ^2^	
		7 day	30 days
2:1	20	91.6 ± 3.9 ^b^	90.3 ± 3.1 ^ab^
	30	92.7 ± 2.1 ^ab^	86.5 ± 2.2 ^bc^
	40	91.6 ± 3.0 ^b^	83.7 ± 3.9 ^cd^
1:1	20	95.7 ± 3.1 ^a^	92.0 ± 2.8 ^a^
	30	95.9 ± 0.7 ^a^	92.6 ± 1.3 ^a^
	40	95.0 ± 2.0 ^a^	87.1 ± 1.2 ^bc^
Free carvacrol ^1^		84.1 ± 1.1 ^c^	80.0 ± 1.8 ^d^

^1^ Free carvacrol was prepared in ethanol at the same concentration as SLNs with a carvacrol loading at 30% mass of lipids. ^2^ Data not sharing the same superscript letter in the same column are significantly different (*p* < 0.05).

**Table 4:** Antimicrobial activities of Car-SLNs with a carvacrol loading at 30% mass of lipids, free carvacrol, and two bare SLNs.

PGMP:GMS	MIC (mg/mL)		MBC (mg/mL)	
	*E. coli* O157: H7	*S. aureus*	*E. coli* O157: H7	*S. aureus*
2:1, bare SLNs	0.300	0.300	0.350	0.350
2:1, Car-SLNs	0.150	0.100	0.200	0.125
1:1, bare SLNs	0.250	0.250	0.300	0.300
1:1, Car-SLNs	0.125	0.100	0.125	0.100
Free carvacrol	0.250	0.125	0.250	0.125

## Supplementary Materials

The following are available online at https://www.mdpi.com/2079-4991/9/8/1162/s1, Figure S1: ^1^H NMR spectra of PGMP, Figure S2: FT-IR spectra of PGMP, Figure S3: ESI-MS of PGMP.
