# Supplementary material for: Carvacrol Loaded Solid Lipid Nanoparticles of Propylene Glycol Monopalmitate and Glyceryl Monostearate: Preparation, Characterization, and Synergistic Antimicrobial Activity
Source: Nanomaterials (Basel). 2019 Aug 14;9(8):1162. doi: 10.3390/nano9081162 (PMC6723752; doi:10.3390/nano9081162)

Supplementary Materials

Carvacrol Loaded Solid Lipid Nanoparticles of Propylene Glycol Monopalmitate and Glyceryl Monostearate: Preparation, Characterization, and Synergistic Antimicrobial Activity

Junbo He [1,2], Shuangshuang Huang [1], Xiaotao Sun [3], Lijuan Han [1,2], Chao Chang [1,2], Weinong Zhang [1,2,]* and Qixin Zhong [4]

[1] Key Laboratory for Deep Processing of Major Grain and Oil, Ministry of Education, College of Food Science & Engineering, Wuhan Polytechnic University, Wuhan 430023, China

[2] Hubei Key Laboratory for Processing and Transformation of Agricultural Products, Wuhan Polytechnic University, Wuhan 430023, China

[3] Beijing Key Lab of Flavor Chemistry, Beijing Technology and Business University, Beijing 100048, China

[4] Department of Food Science, The University of Tennessee, Knoxville, TN 37996, USA

* Correspondence: zhangweinong@163.com; Tel.: +86-027-8392-4790

Figure S1. ^{1}H NMR spectra of PGMP. ^{1}H NMR (400 MHz, DMSO-d6): 0.85 (t, J = 4.0 Hz, 3H), 1.03 (d, J = 4.0 Hz, 3H), 1.23 (s, 24H), 1.51 (s, 2H), 2.28 (t, J = 4.0 Hz, 2H), 3.78-3.87 (m, 2H).

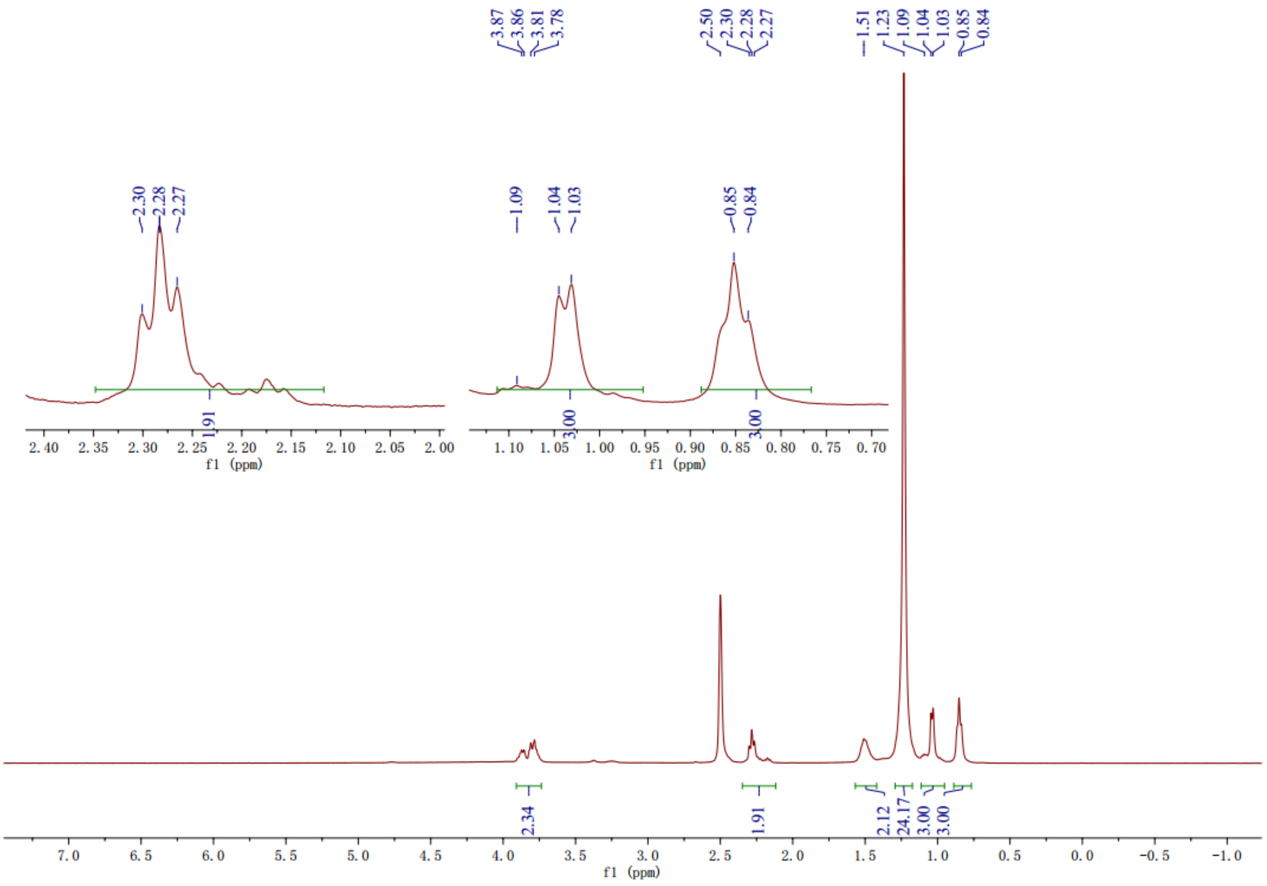

Figure S2. FT-IR spectra of PGMP. FT-IR (KBr, cm^{-1}): 3404 (stretching of OH), 2919 (stretching of alkyl group), 2849 (stretching of alkyl group), 1743 (stretching of C=O), 1460 (bending vibration of alkyl group), 1248 (skeletal vibration of C-C), 1106 (stretching of C-O), 725 (rocking of long C-H group).

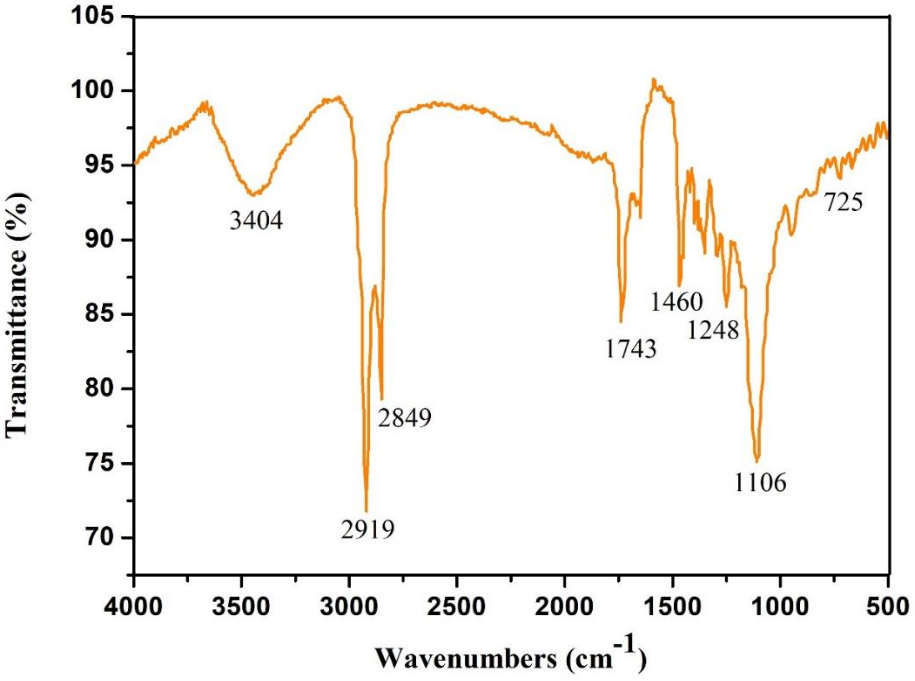

Figure S3. ESI-MS of PGMP. ESI-MS m/z (%): 337.37 ($[M+Na]^+$, 100).

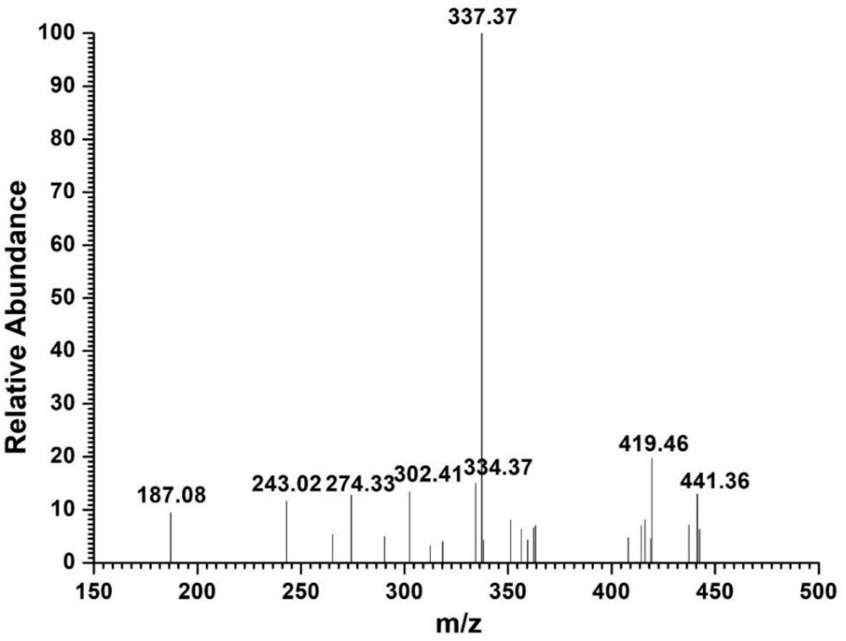

Supplement: Supplementary file 1 [file nanomaterials-09-01162-s001.pdf]
